# Is Ultrasound a Better Target than Clinical Disease Activity Scores in Rheumatoid Arthritis with Fibromyalgia? A Case-Control Study

**DOI:** 10.1371/journal.pone.0118620

**Published:** 2015-03-04

**Authors:** Rafael Mendonça da Silva Chakr, João Carlos Tavares Brenol, Marina Behar, José Alexandre Mendonça, Charles Lubianca Kohem, Odirlei André Monticielo, Claiton Viegas Brenol, Ricardo Machado Xavier

**Affiliations:** 1 Hospital de Clínicas de Porto Alegre, Universidade Federal do Rio Grande do Sul, Porto Alegre, Rio Grande do Sul, Brazil; 2 Pontifícia Universidade Católica de Campinas, Campinas, São Paulo, Brazil; Singapore Immunology Network, Agency for Science, Technology and Research (A*STAR), SINGAPORE

## Abstract

**Objectives:**

Our goal is to study the correlations among gray-scale seven-joint ultrasound score (GS-US7), power Doppler seven-joint ultrasound score (PD-US7), disease activity score-28 joints (DAS28), simplified disease activity index (SDAI) and clinical disease activity index (CDAI) in patients with and without fibromyalgia (FM).

**Methods:**

A matched case-control study included all patients consecutively seen in the Rheumatoid Arthritis (RA) Clinic. Participants were allocated into one of two groups: RA with FM and RA without FM. Ultrasound (US) and clinical scoring were blinded for the presence of FM. Medians and proportions were compared by Mann-Whitney’s test and McNemar’s test, respectively. Spearman’s rank correlation coefficients (r_s_) were calculated among clinical and US scores and differences were tested by r-to-z transformation test.

**Results:**

Seventy-two women were included, out of 247 RA patients, mostly white, with median (IQR) age of 57.5 (49.3–66.8) years, with RA symptoms for 13.0 (6.0–19.0) years and FM symptoms for 6.0 (2.0–15.0) years. Disease-modifying antirheumatic drugs, non-steroidal anti-inflammatory drugs and prednisone use was comparable between groups. Objective activity parameters were not different between groups. RA patients with FM had greater DAS28, SDAI and CDAI but similar GS-US7 and PD-US7. GS-US7 correlated with DAS28, SDAI and CDAI in patients with and without FM (r_s_ = 0.36–0.57), while PD-US7 correlated with clinical scores only in patients without FM (r_s_ = 0.35–0.38).

**Conclusion:**

To our knowledge, this is the first study to demonstrate that ultrasound synovitis scores are not affected by FM in RA patients. PD-US7 performed better than GS-US7 in long-standing RA patients with DAS28, SDAI or CDAI allegedly overestimated due to FM. Since sonographic synovitis predicts erosion better than swollen joint count, C-reactive protein and erythrocyte sedimentation rate, US should be considered a promising treatment target in RA patients with FM.

## Introduction

Rheumatoid arthritis (RA) is a chronic inflammatory disease characterized by erosive synovitis. Synovial *pannus* is the destructive proliferated tissue responsible for bone and cartilage damage. As a *quasi*-malignant tissue, *pannus* becomes thicker and more vascularized, assessed in clinical examination by joint palpation.[1]

RA is treated with disease modifying antirheumatic drugs (DMARDs) to prevent joint destruction. Most importantly, DMARDs should be used under an intensive treat-to-target strategy. Patients must have their disease activity systematically evaluated to adjust their DMARDs treatment. Disease activity may be appraised by clinical composite scores, such as disease activity score of 28 joints (DAS28), simplified disease activity index (SDAI) and clinical disease activity index (CDAI). Each score categorizes disease activity in one of four levels: remission, low, moderate or high. The goal is to achieve remission or low disease activity.[2]

Fibromyalgia (FM) is a chronic pain condition accompanied by somatic symptoms such as fatigue and sleep disorders. FM is present in up to 20% of RA patients and may increase subjective components of disease activity indexes, misleading treatment decision.[3] FM-induced overestimation of RA activity may cause overtreatment as DMARDs are changed to achieve the target and undertreatment as physicians aware of FM impact on clinical scores may not change DMARD when truly necessary.[4]

**Fig 1:**
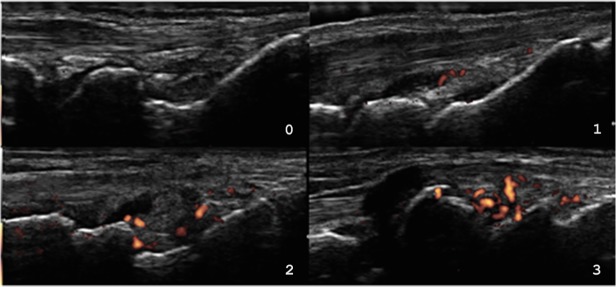
Ultrasound semiquantitative synovitis score. 0: no synovial hypertrophy and no Doppler signal; 1: synovial thickening filling the angle between the periarticular bones, without bulging over the line linking tops of the bones and up to three single Doppler spots signals or up to two confluent spots or one confluent spot + up to two single spots; 2: synovial thickening bulging over the line linking tops of periarticular bones and vessels signals in less than half of the synovium; 3: synovial thickening bulging over the line linking tops of periarticular bones and with extension to at least one of the bone diaphyses and vessels signals in more than half of the synovium.

Ultrasound (US) is an objective synovitis assessment method. US is more sensitive than clinical examination and predicts joint destruction. Gray-scale (GSUS) and power Doppler ultrasound (PDUS) are capable of measuring synovial proliferation and vascularization, respectively.[5] At the joint level, GSUS and PDUS measure the synovitis according to a validated semiquantitative score as 0, 1, 2 or 3 (Fig. 1).[6, 7] At the patient level, several US scores have been proposed to globally assess RA.[7] The validated 7-joint score (US7) combines each joint score for synovial proliferation (GS-US7) and vascularization (PD-US7).[6] As an objective method, US7 is supposed to overcome the subjectivity of DAS28, SDAI and CDAI, but, form the best of the authors knowledge, this has not been demonstrated yet in RA with FM.

Our goal is to study the correlations among GS-US7, PD-US7, DAS28, SDAI and CDAI in patients with and without FM.

## Materials and Methods

A cross-sectional matched case-control study nested in a cohort of RA patients was conducted from March 2012 through March 2013. Recruitment was conducted until the estimated sample size was reached. All participants were consecutively recruited from the cohort and were allocated into one of two groups: RA with FM (cases) and RA without FM (controls). Participants’ allocation was performed after the study visit, when inclusion and exclusion criteria and protocol procedures were applied. Inclusion criteria were RA and FM according to 2010 American College of Rheumatology classification criteria [8, 9], female gender, and age of arthritis onset ≥ 18 years. Exclusion criteria were concomitant systemic inflammatory conditions, hospital admission, surgery, joint infiltration and infections four weeks prior to study visit, pregnancy, and lactation. Controls were recruited from the same cohort and were paired to cases by disease duration and rheumatoid factor positivity. The research protocol was approved by Hospital de Clínicas de Porto Alegre ethics committee and all participants signed informed consent form before entering the study in compliance with the Helsinki Declaration.

US examination was performed by an experienced rheumatologist following international guidelines in a MyLab50 (Esaote do Brasil, São Paulo, Brazil), B mode frequency of 18MHz, gain 60–80%, and power Doppler frequency 6.6–8.0MHz, gain at noise threshold, PRF 0.5–0.75KHz, WF low. Every US exam was run in the morning in a temperature-controlled room (73.4°F or 23°C) after a 30-minute smoking-free rest. Images were saved in high quality for posterior scoring. US7 evaluates inflammatory (synovitis and tenosynovitis) and erosive changes in the wrist, second and third metacarpophalangeal (MCP) and proximal interphalangeal (PIP), and second and fifth metatarsophalangeal (MTP) joints in the dominant side, therefore including the sites that are most affected by RA.[6, 7] For synovitis estimation, wrist and hand joints are assessed in their dorsal, ulnar and palmar aspects, whereas foot joints are only scanned dorsally. During the exam, a total of 13 PDUS and 9 GSUS images are obtained per patient (as dorsal MCP II, III and dorsal PIP II, III images are only in PDUS).[6] The semiquantitative score adopted here was originally used in the US7 study and has been widely validated in other synovitis appraisal scores. [6, 7, 10] In the semiquantitative score, each GSUS and PDUS image is scored 0, 1, 2 or 3, according to its degree of synovial proliferation and vascularization, respectively (Fig. 1).[6, 7, 10] The maximum GS-US7 and PD-US7 per patient are 27 and 39, respectively.[6]

Clinical examination consisted of fibromyalgia impact questionnaire (FIQ), DAS28, SDAI, CDAI and health assessment questionnaire (HAQ), and was performed by an experienced rheumatologist blinded for FM status. Laboratory, medical and demographic data were obtained from electronic records and standardized research forms.

US images acquisition was blinded for RA clinical disease activity level, but blinding for FM diagnosis was not possible due to inevitable patient-examiner interaction during the test. US7 scoring was performed after US examination by a blinded rater of all anonymous randomly assorted high-quality saved images. Intrarater and inter-rater GS-US7 and PD-US7 agreement *kappa* coefficients were calculated both for images acquisition and for still images analyses. Intrarater reliability was calculated for six random patients. A sample of 30 images representing the whole spectrum of synovitis was used for inter-rater reliability. An experienced rheumatologist blinded for RA activity level and FM diagnosis collaborated in inter-rater reliability measuring.

Statistical analyses were performed in WinPepi 11.35 and SPSS 20.0. Each variable was tested for normality (Shapiro-Wilk’s test) and homoscedasticity (Fisher’s *F* test) and, when indicated, results were presented as medians (interquartiles range, IQR) and proportions and between groups differences were tested by Wilcoxon’s *T* test and McNemar’s test, respectively. Spearman’s rank correlation coefficients (r_s_) were calculated among DAS28, SDAI, CDAI, GS-US7 and PD-US7 for each group and differences were tested by r to z transformation test with respective 95% confidence intervals (95%CI). Correlations coefficients previously reported in the literature varied between 0.3 and 0.7 (variation: 0.4).[7, 11, 12] Thus, as no reference study was found for sample size calculation, we used a difference of 0.4 as clinically significant. A sample size of 72 (36 per group) was calculated using r to z transformation test with power of 0.80 to detect a 0.40 difference between groups correlation coefficients. A *P* value of less than 0.05 (two-tailed) was considered significant.

## Results

Seventy-two women were included out of 247 RA patients seen in a University-based RA Clinic. They were mostly White, with median (IQR) age of 57.5 (49.3–66.8) years, had RA symptoms for 13.0 (6.0–19.0) and cases reported FM symptoms for 6.0 (2.0–15.0) years (Table 1).

**Table 1:** Clinical and laboratory characteristics of groups.*

	RA with FM (n = 36)	RA without FM (n = 36)	*P* value^¶^
Demographic			
Age (years)	57.5 (50.0, 66.5)	57.5 (48.3, 66.8)	0.62
White (%)	97	81	0.07
Education level ≤ 4 years (%)	69	69	1.00
Medical			
Sjögren’s syndrome (%)	17	8	0.45
Fibromyalgia			
FM duration (years)	6.0 (2.0, 15.0)	-	-
Tender points (0–18)^§^	14.0 (12.0, 16.75)	4.0 (2.0, 6.0)	<0.001
FIQ (0–100) ^§^	56.8 (40.5, 74.7)	-	-
FIQ pain (0–10) ^§^	6.1 (1.9, 9.5)	-	-
Pain medications† use (%)	64	22	<0.001
Rheumatoid arthritis			
RA duration (years)	12.5 (6.0, 19.0)	13.0 (6.0, 17.8)	0.60
Rheumatoid factor (+) (%)	78	78	1.00
Erosion on radiograph (%)	64	69	0.73
Conventional DMARD use (%)	97	97	1.00
Biologic DMARD use (%)	19	17	0.82
NSAID use (%)	9	22	0.13
Prednisone dose (mg/day)	1.5 (0.0, 10.0)	2.5 (0.0, 10.0)	0.61
HAQ (0–3) ^§^	1.6 (0.9, 2.1)	0.9 (0.4, 1.3)	<0.01
DAS28 (0–9.4) ^§^	5.2 (4.3, 6.3)	4.0 (3.3, 4.6)	<0.001
SDAI (0–86) ^§^	31.1 (18.0, 40.3)	13.1 (9.0, 22.4)	<0.001
CDAI (0–76) ^§^	30.4 (18.0, 39.7)	13.1 (8.0, 22.1)	<0.001
Tender joints count (0–28) ^§^	14.0 (7.0, 22.8)	2.5 (1.0, 6.0)	<0.001
Swollen joints count (0–28) ^§^	5.0 (1.3, 8.0)	3.0 (2.0, 6.0)	0.25
Patient visual analog scale (0–100) ^§^	59.5 (39.5, 76.8)	38.0 (17.3, 59.3)	<0.01
Erythrocyte sedimentation rate (mm/h)	12.5 (7.0, 31.0)	19.5 (9.0, 32.8)	0.16
C-reactive protein (mg/l)	10.1 (0.0, 13.6)	5.1 (4.0, 11.0)	0.14
GS-US7	10.0 (7.0, 11.0)	9.0 (7.0, 11.0)	0.37
PD-US7	3.0 (1.0, 5.8)	4.0 (2.0, 5.0)	0.87
Synovial hypertrophy on US (%)	100	100	1.00
Synovial effusion on US (%)	33	44	0.34

RA: rheumatoid arthritis; FM: fibromyalgia; FIQ: fibromyalgia impact questionnaire; FIQ pain: “How much pain did you experience in the last seven days?”; RA: rheumatoid arthritis; DMARD: disease-modifying antirheumatic drugs; NSAID: non-steroidal anti-inflammatory drugs; mg: milligrams; HAQ: health assessment questionnaire; DAS28: disease activity score of 28 joints with 4 variables (tender joints count, swollen joint count, patient visual analog scale, erythrocyte sedimentation rate); SDAI: simplified disease activity index with 5 variables(tender joints count, swollen joint count, patient visual analog scale, physician visual analog scale, C-reactive protein); CDAI: clinical disease activity index with 4 variables (tender joints count, swollen joint count, patient visual analog scale, physician visual analog scale); GS-US7: gray-scale 7-joint ultrasound score; PD-US7: power Doppler 7-joint ultrasound score; US: ultrasound.

*Values are median (25^th^, 75^th^ percentiles) or percentage

^¶^Two-tailed Wilcoxon’s or McNemar’s tests; *P*<0.05

^§^Score (range)

†Pain medications: tricyclic antidepressants, selective serotonin reuptake inhibitors, cyclobenzaprine, duloxetine, pregabalin or tramadol

Median FIQ was 56.8 (40.5–74.7) with a FIQ pain scale of 6.1 (1.9–9.5) among RA patients with FM. The number of tender points was 14.0 (12.0–16.5) among RA patients with FM and 4.0 (2.0–6.0) in RA patients without FM. RA patients with FM used more often pain medications than those without FM. DMARDs, non-steroidal anti-inflammatory drugs and prednisone use was comparable among RA patients with and without FM.

Also, objective inflammatory parameters were not different between groups. RA patients with FM had greater DAS28, SDAI and CDAI than those without FM, but similar GS-US7 and PD-US7 (Table 1). GS-US7 varied from 1 through 20 in RA patients with FM and from 3 through 17 in patients without FM. PD-US7 varied from 0 through 20 in patients with FM and from 0 through 12 in patients without FM.

All agreement kappa coefficients were statistically significant (*P*<0.001). For GS-US7 and PD-US7 image acquisition, inter-rater coefficients were 0.56 and 0.72, and intrarater coefficients were 0.64 and 0.70, respectively. For GS-US7 and PD-US7 still image scoring, inter-rater coefficients were 0.50 and 0.66, and intrarater coefficients were 0.54 and 0.67, respectively.

GS-US7 correlated with DAS28, SDAI and CDAI in RA patients with and without FM (Table 2). Although numerically greater in patients without FM, correlation coefficients were not statistically different between groups. PD-US7 correlated with clinical scores only in RA patients without FM; no statistically significant coefficients were found in patients with FM (Table 2). A close to significance difference between groups was found for PD-US7, SDAI and CDAI coefficients.

**Table 2:** Spearman’s correlations coefficients among US and clinical scores.

Variables	Correlation in RA with FM (n = 36)	Correlation in RA without FM (n = 36)	Difference (95%CI) ^¶^
GS-US7 and DAS28	0.36*	0.39*	-0.03 (-0.44 to 0.38)
GS-US7 and SDAI	0.38*	0.57*	-0.19 (-0.56 to 0.17)
GS-US7 and CDAI	0.43*	0.57*	-0.14 (-0.50 to 0.22)
PD-US7 and DAS28	0.12	0.35*	-0.23 (-0.65 to 0.21)
PD-US7 and SDAI	0.01	0.38*	-0.37 (-0.78 to 0.08)
PD-US7 and CDAI	0.01	0.37*	-0.36 (-0.77 to 0.09)

* *P*<0.05

^¶^ r to z transformation test

DAS28: disease activity score of 28 joints with 4 variables (tender joints count, swollen joint count, patient visual analog scale, erythrocyte sedimentation rate); GS-US7: gray-scale 7-joint ultrasound score; PD-US7: power Doppler 7-joint ultrasound score.

The correlations among GS-US7, PD-US7 and FIQ were not significant (r_s_ = -0.4 and 0.01, respectively; *P*>0.05). However, both ultrasound scores correlated to tender joints count (TJC) in RA patients without FM (GS-US7, r_s_ = 0.37; PD-US7, r_s_ = 0.35; *P*<0.05). In RA patients with FM, only GS-US7 correlated to TJC (GS-US7, r_s_ = 0.34, *P*<0.05; PD-US7, r_s_ = 0.02, *P*>0.05).

## Discussion

To our best knowledge this is the first study to demonstrate that ultrasound synovitis scores are not affected by FM in RA patients. In addition, PD-US7 and clinical scores correlated only in patients without FM, indicating that PD-US7 may better assess disease activity than GS-US7 in RA patients with FM.

We have previously demonstrated that swollen joint count (SJC), erythrocyte sedimentation rate (ESR) and C-reactive protein (CRP) are not affected by FM in RA patients.[3] As objective measures, SJC, ESR and CRP help identify RA patients with clinical disease activity overestimation due to FM. However, none of these measures has been individually validated as an erosion predictor. Besides its objectivity, sonographic synovitis is a validated erosion predictor and should be considered a better measure to follow RA patients with FM.[5, 6] As recently demonstrated, US modified treatment decision in 29% of DAS28-based assessments.[13] The impact of US in RA with FM could be even greater, when mistreatment is expected to be more frequent.[4]

In the present study, RA patients with FM had significantly higher values of tender joints count and patient visual analog scale; however, a similar number of swollen joints count was observed. These findings indicate that RA patients with and without FM apparently had the same level of objective synovitis, but perceived their arthritis differently. In a larger study by Ranzolin *et al* RA patients with FM (n = 238) had greater tender joints count and patient visual analog scale (*P*<0.001) than RA patients without FM (n = 32). In this prospective cross-sectional study, RA patients with and without FM had similar swollen joints count (*P* = 0.119) and erythrocyte sedimentation rate (*P* = 0.343). [3]

Out of the several validated ultrasound scores, including different joints, we opted for the US7, which comprehends the regions mostly affected by RA. [6] Despite including only small joints of one hand and one foot, the US7 fulfills the Outcome Measures in Rheumatology (OMERACT) filter of truth, discrimination and feasibility.[6, 14, 15] The US7 is as accurate as other validated ultrasound scores with a fairly small number of joints to be assessed, becoming an interesting global ultrasound scoring system of synovitis in RA at the patient level. [7, 15–17]

The strength of correlations between clinical and ultrasound scores in the group without FM was comparable to the existing literature.[7, 11, 12] GS-US7 and clinical scores varied accordingly in both groups. GS-US7 and SJC correlated irrespective to FM status, as they measure the same phenomenon (synovial proliferation). SJC is not influenced by FM and integrates DAS28, CDAI and SDAI.

Also, PD-US7 and clinical scores varied accordingly in patients without FM, but not in FM patients. In the study by Damjanov *et al*., PDUS-based joint count and TJC correlated, insinuating PDUS as a surrogate for tenderness.[18] In FM, pain is not necessarily caused by arthritis, as central sensitization may explain tenderness even when there is no synovitis. In RA patients with symptomatic FM, PD-US7 (synovial vascularization) and TJC (pain sensitivity) are assessing different phenomena and, therefore, do not correlate. Since the correlations among PD-US7 and overestimated clinical scores were nullified by FM, we understand that PD-US7 is better than GS-US7 to assess RA activity in these patients.

Our study identified a difference of 0.30 between groups’ coefficients as a reference for future studies. As blinding for FM status was not possible during US exam, all 1,584 anonymous images were blindedly scored after the completion of study. Besides, acceptable agreement coefficients obtained from blinded examiners were similar to previous studies. [14, 17]

## Conclusions

In conclusion, GS-US7 and PD-US7 were similar in patients with and without FM. PD-US7 correlated with clinical disease activity scores only in patients without FM. Probably, PD-US7 better identifies long-standing RA patients with DAS28, SDAI or CDAI overestimated due to FM. Since sonographic synovitis predicts erosion better than SJC, CRP and ESR, US should be considered a promising treatment target in RA patients with FM. Ultimately, longitudinal studies comparing sonographic and clinical scores may demonstrate how each target improves the value of DMARDs use in RA with FM.
